# A Three-Lesson Teaching Unit Significantly Increases High School Students’ Knowledge about Epilepsy and Positively Influences Their Attitude towards This Disease

**DOI:** 10.1371/journal.pone.0150014

**Published:** 2016-02-26

**Authors:** Uwe K. Simon, Lisa Gesslbauer, Andreas Fink

**Affiliations:** 1 Center for Didactics of Biology, Karl-Franzens-University Graz, Schubertstraße 51, 8010, Graz, Austria; 2 Institute of Psychology, Karl-Franzens-University Graz, Universitätsplatz 2/DG, 8010, Graz, Austria; University of Rome Tor Vergata, ITALY

## Abstract

Epilepsy is not a regular topic in many countries’ schools. Thus many people harbor misconceptions about people suffering from this disease. It was our aim to a) examine what grade ten students know and believe about epilepsy, and b) to develop and test a teaching unit to improve their knowledge and attitude. The test group comprised eight grade ten classes from six different Austrian high schools (54 girls and 51 boys aged 14–17), the control group (no intervention) five grade ten classes from the same schools (26 girls and 37 boys aged 14–17). The teaching unit consisted of three 45-min lessons using different methods and material. Changes in knowledge about and attitude towards epilepsy as a result of the intervention were psychometrically assessed in a pre-test intervention post-test design (along with a follow-up assessment two months after the intervention) by means of a questionnaire capturing different facets of epilepsy-related knowledge and attitude. Across all knowledge/attitude domains, students of the test group had a significantly improved knowledge about and a more positive attitude towards epilepsy and people suffering from it after the teaching unit. However, starting levels were different between the five knowledge/attitude domains tested. Medical background knowledge was lowest and consequently associated with the highest increase after the intervention. This study shows that epilepsy-related knowledge of many grade ten high school students is fragmentary and that some harbor beliefs and attitudes which require improvement. Our comprehensive but concise teaching unit significantly increased knowledge about epilepsy and positively influenced attitude towards individuals with epilepsy. Thus we recommend implementing this unit into regular school curricula.

## Introduction

Up to one per cent of the world’s population suffers from epilepsy making it one of the most frequent neurological diseases [1]. In Austria there are about 65,000 people with one or another form of epilepsy [2], which is roughly 0.8 percent of the total population. Consequently, about every 125^th^ Austrian may develop epileptic symptoms in his or her lifetime. Approximately 30 per cent of all people with epilepsy show symptoms already during childhood [3]. However, there are many studies showing that individuals with epilepsy are often confronted with misconceptions about this disease [4–9].

Thus there is urgent need to introduce pupils to this disease and to study children’s and adolescents’ attitudes towards and knowledge about epilepsy. But epilepsy is not a regular topic in Austrian (and German) school curricula. As a consequence, knowledge about this disease relies solely on individual teachers’ efforts which usually results, if at all, in single-lesson presentations often prepared by students. Yet such a widespread disease, which furthermore demands precise knowledge about what to do during a seizure, requires more attention at school. From a social perspective, it is important to help individual students with epilepsy to overcome their fear that classmates may treat them unfriendly because of their perceived strange and uncontrolled behaviour. To know that their surrounding is aware of why seizures occur, how to act in such circumstances and that epilepsy is nothing to be afraid of or to make fun of will be an enormous relief for such children [10,11]. This is highly important, since the integration of people with epilepsy into public life is apparently still far from acceptable [12,13]. Many studies show that a minor but still relatively large proportion of a given nation’s population has misconceptions about epilepsy and has little knowledge about this disease [6,7,14–16].

It was the aim of the present study to investigate what teenagers at Austrian high schools know and believe about epilepsy, and whether a concise but intensive mixed-method intervention during regular biology classes would lead to changes in their knowledge and attitude. We chose to develop and test school material for grade ten students for two reasons: a) Attitudes and perceptions are mostly formed during the developmental years making this age group or younger students the appropriate target, and 2) the nervous system and modern brain research are topics in upper secondary schools in Austria, usually in grade ten [17].

## Materials and Methods

### Teaching material and test group

Several interest groups offer fact sheets concerning epilepsy, in particular to inform teachers how to deal with students with epilepsy [10,18,19]. However, to our knowledge there is virtually no teaching material available in German, which deals with epilepsy itself in sufficient depth and could directly be used at school. Consequently, we developed material for grade ten students and tested its effects with eight classes from six Austrian high schools in Styria, taught by the same instructor (first author) in the school year 2014/15. (The material will be freely available in German language at the webpage of the German teacher magazine MNU [20]: http://www.mnu.de/zeitschriften/.) Schools were chosen based upon willingness of teachers to participate. Overall, 168 students took part in this study: The test group consisted of 54 girls and 51 boys aged 14–17 (mean age 15.35, SD 0.63), the control group of five grade ten classes from the same schools (26 girls, 37 boys aged 14–17; mean age 15.41, SD 0.66). Students who did not attend all lessons were not included in these figures. Permission for this study was given by the federal states’ school authority. Since the federal states’ school authority is responsible for studies involving teaching interventions at school and student questionnaires this institution was asked for permission and not the university’s ethics commission. Parents, schoolmasters, and teachers were informed prior to study begin by an information sheet and asked for consent. The information sheet for participant's parents was distributed by the respective biology teacher of the class. Parents were asked to notify teacher or researcher, if they did not want their child to take part in the study, which did not happen in a single case. Thus, all children from all classes were granted participation by their parents. To our knowledge, one male student in the control group suffered from epilepsy, but neither we nor his classmates knew his identity. His participation was granted by both his parents and the teacher.

### Timescale

Knowledge and attitude were measured before (pre-) and after (post-/follow up-test) the intervention using a questionnaire capturing different facets of epilepsy-related knowledge and attitude (S1 Material). The pre-test took place about a week before the intervention started. The teaching unit encompassed three regular and consecutive biology lessons with 45 minutes each. The post-test was conducted one week after the final lesson in each class, the follow-up test after another two months.

### Intervention

Using several different teaching methods the participants encountered various facts about epilepsy [20]. Since almost all students had noted “seizure” or “convulsion” when asked for key words they would associate with epilepsy in the pre-test, the intervention started with a discussion about an anonymous quote from a youtube site about seizures. Students were then told facts about epilepsy (medical background information, possible external causes for seizures). Afterwards, students completed a worksheet about seizures. The first lesson ended with tracks from a video about different kinds of seizures (Epilepsie & Arbeit—Gemeinnützige Beratungs- und Entwicklungs-GmbH: “Epileptische Anfälle—Richtiges Verhalten und Erste Hilfe“; only available in German) and a discussion about worksheet results and the video [20].

The second lesson began with a short recall phase and a detailed analysis of the four different seizures types previously seen in the videos. Thereafter students read about and discussed pair-wise electroencephalography and magnetic resonance tomography for diagnosis of epilepsy. They were introduced to famous past and present state leaders, artists, writers, scientists and sportsmen with epilepsy and watched short documentary clips about a sportsman and physiotherapist, a pregnant woman and a teacher all having epilepsy [20] (Deutsche Gesellschaft für Epileptologie e.V.: “Epilepsie leben und Epilepsie verstehen—Erfahrungen von Betroffenen, praktische Informationen, Medizinisches Wissen“; only available in German).

In lesson three students studied extracts from internet blogs discussing therapeutic options (medical treatment or surgery) and were asked to analyse problems for correct medication and the advice offered in the forum. After discussing results students played a quiz using power point slides about first aid for people suffering from a seizure. The unit ended with a short discussion about the aspects of epilepsy dealt with in class [20].

All teaching material and methods were previously discussed amongst first and second author, who are both biology teachers. To check whether students felt comfortable with the teaching unit the post-test also contained questions to find out how interesting students found the topic, whether or not they felt it easy to follow, whether or not the material itself was easy to understand, whether they had missed anything, and what they felt they had learnt. Additionally, we asked whether they believed that their attitude towards people with epilepsy had changed. Since all these items were almost unanimously answered positively the material and its presentation can be regarded as suitable for this age group [20].

### Questionnaires

Items were partly designed on the basis of previously published questionnaires [7,12,21–24]. In these studies, knowledge and attitude regarding epilepsy were assessed for Swiss and German adults and teenagers, Asian students and Italian teachers. Other items were specifically constructed for this study. In almost all cases questions could be answered alternatively with “yes“, “no“, or “I am not sure “.

Items were belonging to four knowledge domains and one attitude domain (Table 1). Items from different domains were mixed to avoid routine answering during completion of the questionnaire. Additionally, we asked whether students had already observed a seizure or knew someone suffering from epilepsy, and whether they would feel uncertain when meeting someone with epilepsy (see S1 Material). Questionnaires were coded by students using the first two letters of the participants’ and their mothers’ and fathers’ names to allow for both anonymity and linking of pre-/post-/follow up-test results. The questionnaires were coded by the students without revealing identity to the researchers.

**Table 1:** Items from the respective knowledge/attitude domain analyzed with ANOVA.

Knowledge about seizures	Knowledge about daily life aspects of epilepsy	Medical knowledge about epilepsy	Stereotypes about epilepsy	First-aid related knowledge concerning epilepsy
*Is epilepsy the same as a seizure*?	*Can a person*, *who has a seizure now and then*, *work as a teacher*?	*Is it possible to diagnose epilepsy using electroencephalography (EEG)*?	*Is epilepsy a mental illness*?	*Should you always immediately call the ambulance in case you observe a seizure*?
*Is ‘loss of consciousness’ a possible symptom of a seizure*?	*Can a person*, *who has a seizure now and then*, *work as a lorry driver*?	*Can an operation help people with epilepsy*?	*Should people with epilepsy be allowed to marry*?	*Should you place soft cushions below a person having a seizure*?
*Is the origin for a generalized seizure epileptic activity in one half of the brain*?	*Are people with epilepsy able to live as self-dependent as people without epilepsy*?	*Is it possible to diagnose epilepsy using magnetic resonance tomography (MRT)*?	*Are people with epilepsy unpredictable and dangerous*?	*Should you place a hard object in the mouth of someone having a seizure to protect the tongue*, *or hold apart the jaw*?
*Is it always possible to recognize a seizure*?	*Can a person*, *who has a seizure now and then*, *work as a metal worker*?	*Is epilepsy generally a heritable disease*?	*Should women with epilepsy be allowed to become pregnant*?	*Should you wet the face of someone who has a seizure*?
*Can someone having had a generalized seizure usually not recall this experience*?	*Are children/ adolescents with epilepsy able to attend regular schools*?	*Can medication usually help people with epilepsy*?	*Are people with epilepsy less intelligent*?	*Should you put a person*, *who has had a seizure with convulsion and loss of consciousness*, *into recovery position*?
*Is ‘convulsion/ twitching of single body parts’ a possible symptom of a seizure*?	*Are people with epilepsy allowed to do sport*?	*Do people with epilepsy always need a lifelong treatment*?	*Can teachers expect children with epilepsy to perform equivalently to those without epilepsy*?	*Should you firmly hold a person having a seizure*?
*Do seizures happening with different people always look the same*?	*Can a person*, *who has a seizure now and then*, *work as a nurse*?		*Would you begin a love relationship with someone you know has epilepsy*?	*Should you remove sharp objects*, *edged furniture etc*., *when you observe a seizure*?
*Is ‘absurd behavior’ a possible symptom of a seizure*?	*Can a person*, *who has a seizure now and then*, *work as a police officer*?			*Should you try to help a person lay down*, *if he/she seems to get a seizure soon*?
*Can a head injury cause a seizure*?				
*Do people with epilepsy always have seizures regularly*?				
*Is ‘convulsion/ twitching of the whole body’ a possible symptom of a seizure*?				
*Is ‘temperature‘ a possible symptom of a seizure*?				
*Is ‘falling down‘ a possible symptom of a seizure*?				
*Can shortage of sleep cause a seizure*?				
*Can extensive drinking of alcohol cause a seizure*?				

Reliability was tested in a pilot study with two Styrian grade ten biology classes (N = 43) of a high school different to the schools used for the main study. Students worked on the questionnaire twice with an inter-test interval of two weeks, and did not receive any epilepsy-related information in between. Re-test-reliability coefficients for the five domains were as follows: domain 1 (seizure-related knowledge): 0.83; domain 2 (daily life-related knowledge): 0.56; domain 3 (medical knowledge): 0.74; domain 4 (stereotypes): 0.69; domain 5 (first aid-related knowledge): 0.79. Thus, all domains were regarded as sufficiently reliable. The somewhat lower correlations of domains 2 and 4 may be due to the fact that students might have been more emotionally involved here (e.g. in questions referring to the possibility to attend regular schools for children suffering from epilepsy, or to pregnancy and loving relationships).

For the main study, students in both the test and the control group were told that any questions concerning the items (except in case of non-understanding) would be answered after the follow-up test. Thereby we wanted to reduce the chance that students would read literature or research the internet and thus get additional information beyond what was discussed during the teaching unit (test group) or at all (control group)–which almost no student did according to self-report in the follow-up test.

### Statistical analyses

In order to investigate the efficacy of the intervention, we used a general linear model (GLM) for repeated measures on the number of correct responses in the questionnaire in considering TIME (3 levels: pre-test, post-test, follow-up) and knowledge/attitude DOMAIN (5 domains, see above) as within-subjects factors and experimental group (test group, controls) as between subjects factor. In case of violations of sphericity assumptions, the multivariate approach to repeated measurements variables was used [25]. Specific post hoc comparisons of means were conducted using the most conservative Scheffé test. The probability of a Type-I-error was kept at *p* < .05 in all statistical analyses.

All data used for statistical analyses can be found in S2 Material.

## Results

### Student previous experience with epilepsy

Twenty six students of the test group (24.8%) and 11 students (17.5%) of the control group knew someone with epilepsy. Only few students had already witnessed a seizure: 11 (10.5%) in the test group and 5 (7.9%) in the control group. 26 students (24.8%) of the test group and 18 students (28.6%) of the control group felt uneasy when thinking of meeting someone with epilepsy. Reasons given were almost exclusively lack of knowledge of what to do in case of a seizure. These figures dropped to 11 students (10.5%) in the test group, but remained stable for the control group in the post-test.

### Changes of epilepsy-related knowledge and attitudes in the test and the control group

**Fig 1:**
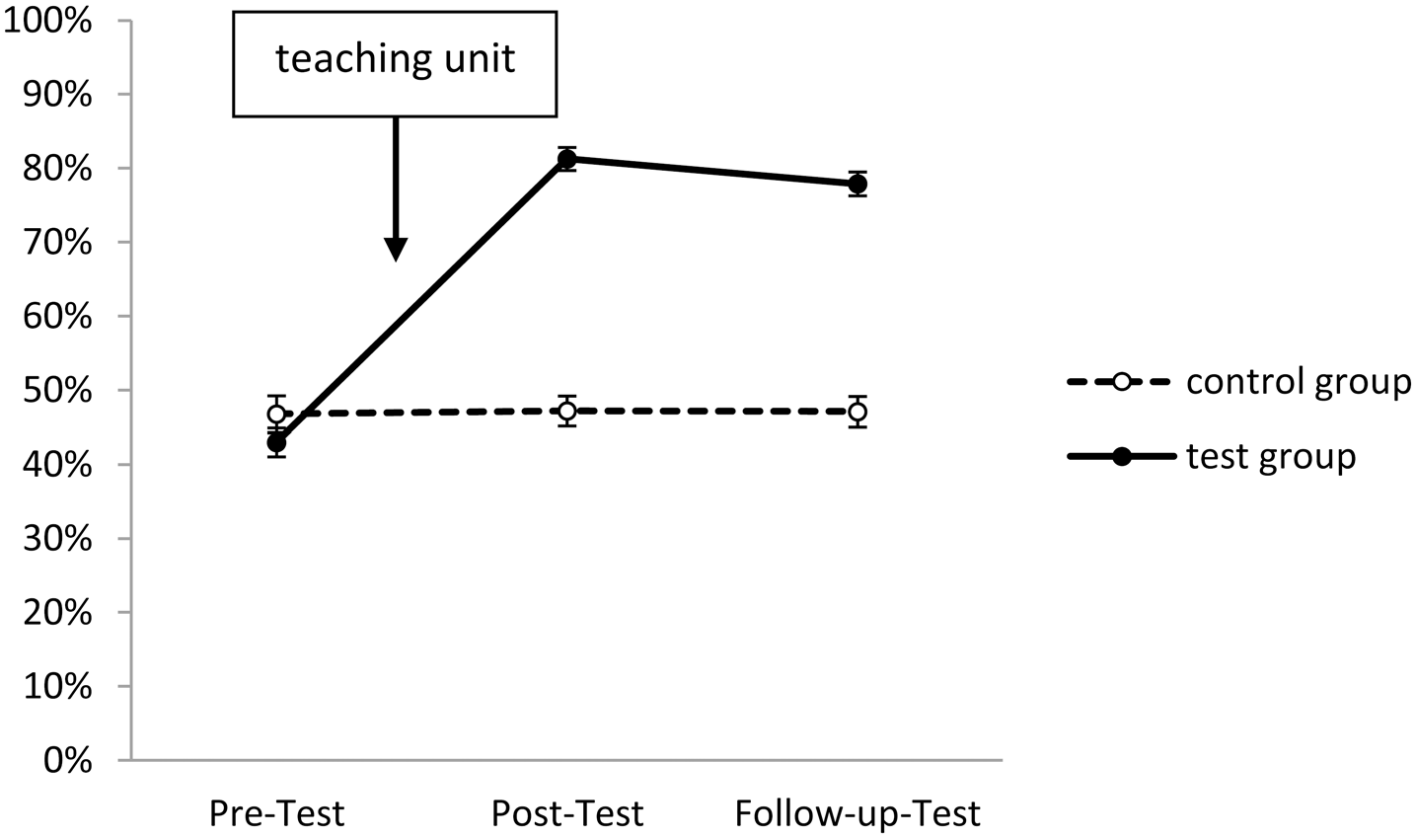
Development of seizure-related knowledge in test and control group (domain 1). Shown are solution rates (mean of all seizure-related items). For descriptives see S1 Table.

**Fig 2:**
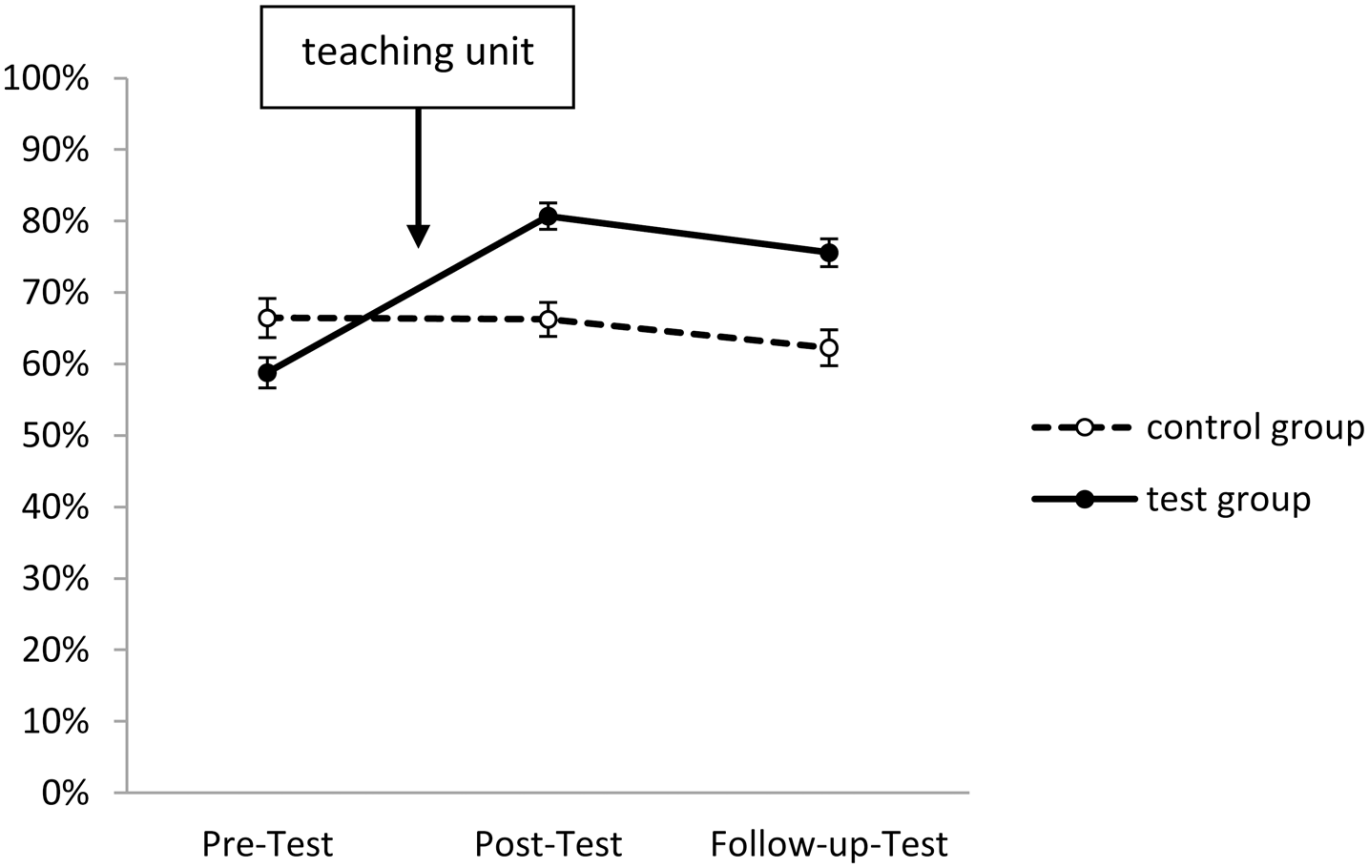
Development of daily life-related knowledge in test and control group (domain 2). Shown are solution rates (mean of all daily life-related items). For descriptives see S1 Table.

**Fig 3:**
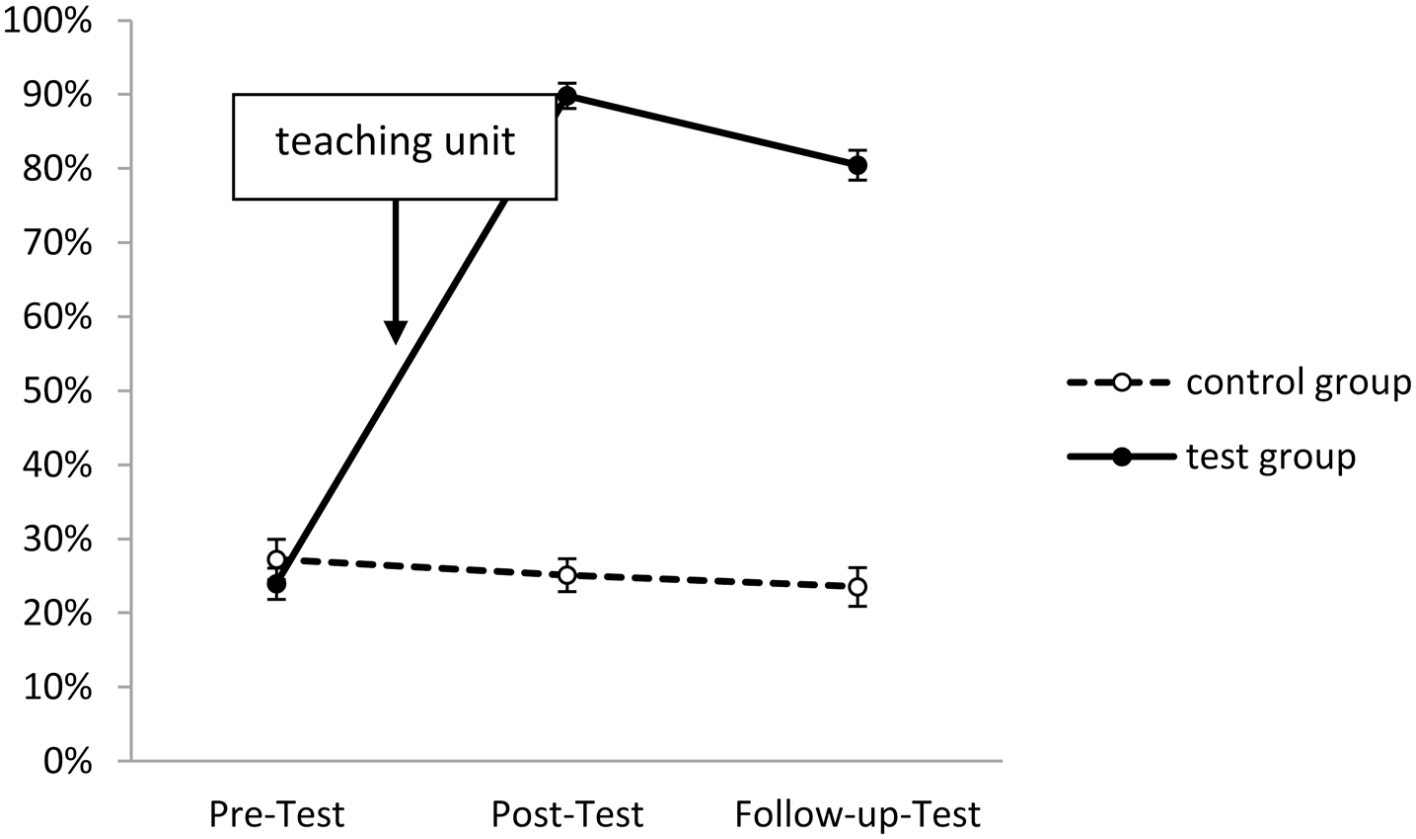
Development of medical knowledge in test and control group (domain 3). Shown are solution rates (mean of all medical knowledge-related items). For descriptives see S1 Table.

**Fig 4:**
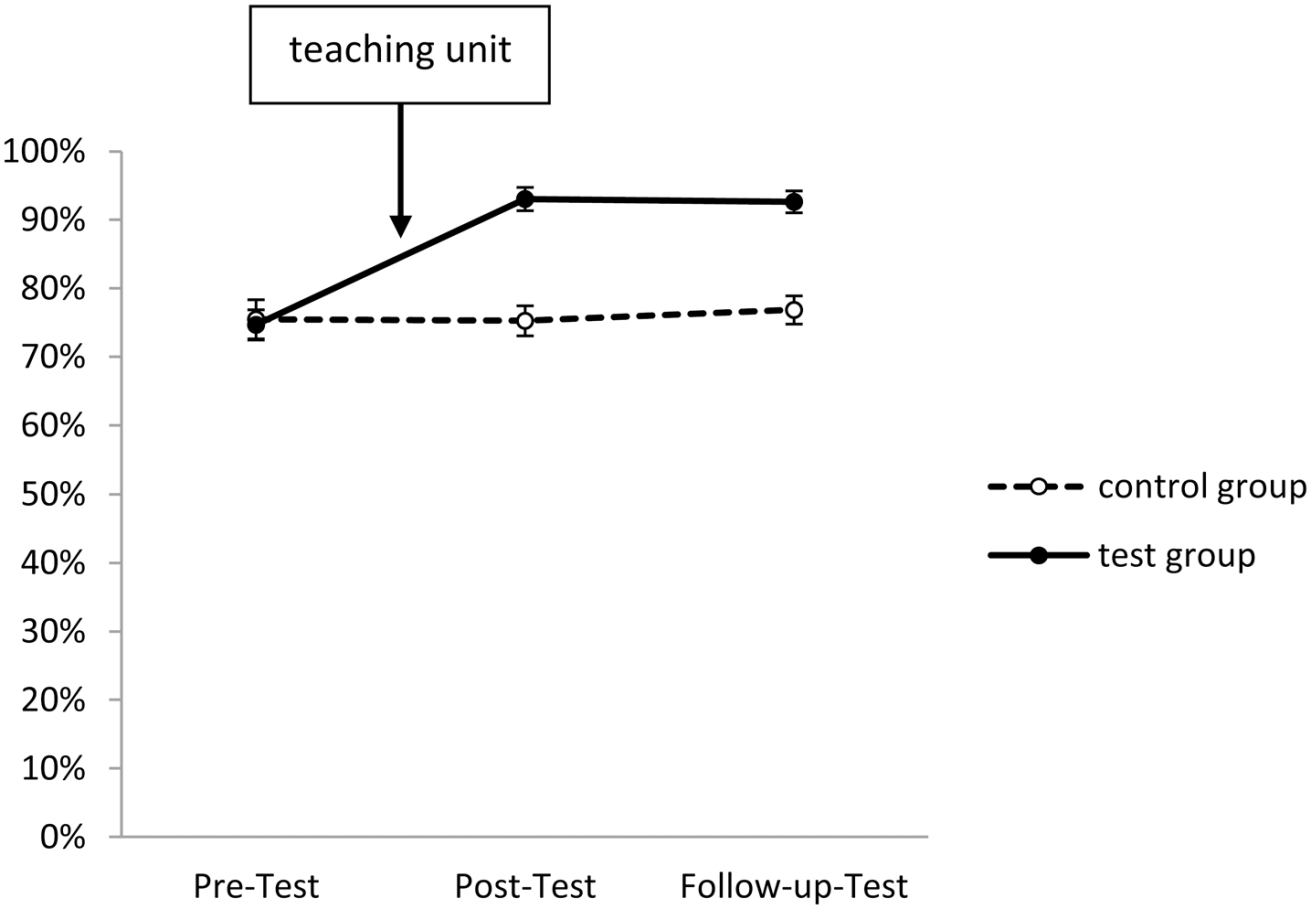
Development of stereotypes in test and control group (domain 4). Shown are solution rates (mean of all stereotype-related items). For descriptives see S1 Table.

**Fig 5:**
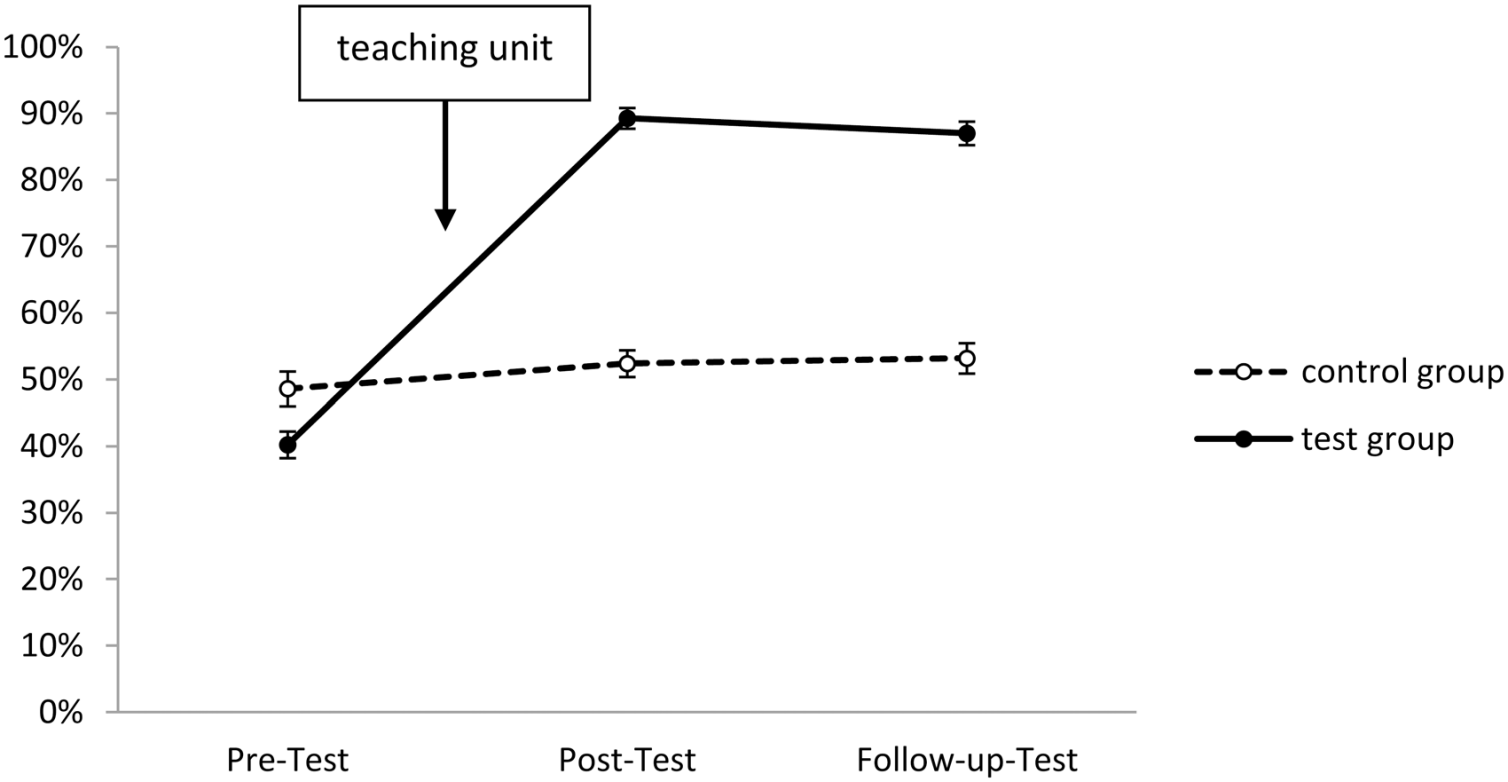
Development of first aid-related knowledge in test and control group (domain 5). Shown are solution rates (mean of all first aid-related-related items). For descriptives see S1 Table.

In Figs 1–5, the solution rates for the epilepsy questionnaire are shown separately for each group, time point of assessment and knowledge/attitude domain. Inspection of these figures revealed that the test group showed considerable increases in each knowledge/attitude domain after the intervention, while there were virtually no changes in the control group. The GLM yielded significant effects for the relevant interactions between TIME and GROUP (*F*(2,165) = 150.41, *p* < .01, *η*^*2*^*p* = .58), and between TIME, DOMAIN and GROUP (*F*(8,159) = 19.28, *p* < .01, *η*^*2*^*p* = .16). Specific post hoc comparisons of means revealed significant (*p* < .05) increases from the pre- to the post-test in the test group in all five knowledge/attitude domains, particularly with respect to medical knowledge, while there were no significant time-related effects in the control group. The intervention effects in the test group remained stable over time, since there were no significant changes in the solution rates from post-test to follow up assessment.

In addition to the relevant interactions involving the factors TIME and GROUP, the main effects TIME (*F*(2,165) = 153.83, *p* < .01, *η*^*2*^*p* = .58), DOMAIN (*F*(4,163) = 169.73, *p* < .01, *η*^*2*^*p* = .56) and GROUP (*F*(1,166) = 122.82, *p* < .01, *η*^*2*^*p* = .43), along with the interactions between DOMAIN x GROUP (*F*(4,163) = 34.39, *p* < .01, *η*^*2*^*p* = .22), and TIME x DOMAIN (*F*(8,159) = 22.83, *p* < .01, *η*^*2*^*p* = .17) were significant. Thus, development for each domain was significantly different to that of all other domains within the test group, and also to those of the control group.

The starting levels of the various domains were partly very different: Slightly less than half of the items about seizure-related knowledge and first-aid related knowledge, about two third of the items concerning daily life related knowledge, but only about one fourth of medical background knowledge related items were correctly answered in the pre-test. Three quarters of attitude-related items were correctly answered (Figs 1–5).

## Discussion

International studies show a wide range of attitudes towards and knowledge about epilepsy. One of the most important predictors seems to be educational status, and, in particular, knowledge about the disease itself. For example, almost 10% of the interviewed Germans (age 15 and above) were negative about people with epilepsy [7]. The same percentage was reported for Austrian adults [6]. In this latter study, “little theoretical knowledge about epilepsy“, “misconception of epilepsy as a form of insanity” and “no personal acquaintance with someone with epilepsy” negatively influenced attitudes of respondents towards people suffering from epilepsy. Based on their results the authors concluded that “knowledge about epilepsy is an independent and even better predictor than is general education” (p. 741). Similar observations come from earlier work from Germany, where the percentage of people regarding epilepsy as a mental illness changed from 27% (1967) to 31% (1973) and 23% (1978) [26, 27], until it reached its hitherto lowest level of 11% in 2008 –which was, however, still far above respective figures for Switzerland or the U.S. [7]. These figures seemed to correlate with educational level of study participants, leading the authors to state that “further education about epilepsy is required, in particular at school”, predominantly via development and spread of educational material and further training of teachers [27].

Our data lend further strength to these statements. Although the attitude towards people with epilepsy was generally rather positive amongst the teenagers participating in our study already at the pre-test, we still observed a positive trend for questions regarding stereotypes after the teaching unit. This implies that the acquisition of epilepsy-related knowledge helped to improve attitude towards this disease.

The most prominent increase could be observed in terms of medical knowledge (see Fig 3), which was also the domain with the lowest starting levels—indicating, that epilepsy-related medical and biological knowledge is very fragmentary amongst people of this age group. For example, only 44 students from the test group denied that epilepsy and seizures are the same in the pre-test, but 97 did so in the post-test and still 94 in the follow-up test, which proves that students had sustainably learned to differentiate between the disease and its many different symptoms. To give another example: In terms of treatment only 6 (5.7%) students of the test group knew or believed in advance that a surgery may successfully heal epilepsy. This figure increased to 97 (92.4%) directly after the teaching unit and 86 (81.9%) in the follow-up test, which is far higher than the 18% reported for the Swiss population [21], or the 10.5% for Italian teachers [24]. These figures show that the intervention tested here indeed lifted knowledge and attitude levels far above those reported in international non-educational surveys.

The number of students who were acquainted with someone suffering from epilepsy and/or had observed a seizure is far below those reported in many other studies (see Table 1 in Spatt et al. 2005 for references), but matches well with results from Austrian adults [6]. The difference between seizure observation and personal acquaintance might be explained by the often infrequent occurrence of the first, by medication, or by patients’ shyness to let their surroundings know about their condition. However, data for Switzerland show that especially younger people were less well informed about this disease in 2011 than in 2003 [21] indicating that there is need for better information at school.

Even though differences between pre- and post-/follow-up tests for the test group, and between test and control group are highly significant, we do not claim that our results are representative for all Austrian adolescents due to the relatively low number of participants and the choice of the student population. For example, further studies would have to analyze whether the same intervention is successful for students not attending high school, and whether the latter are similar or not in their pre-test knowledge and attitudes. On the other hand, pre-test answers from students are very much in line with those for Swiss and German adults [7,21]. Our results gain further strength from the fact that trends were not parallel for all domains tested, which, of course, also originated in the different starting levels for each domain.

Concerning attitude, our figures indicate that the intervention was successful in that almost no student regarded people suffering from epilepsy as less intelligent or erratic and dangerous after the intervention. The same is true for questions concerning marriage and pregnancy. Here, the overall extremely positive answers in all tests by far exceeds figures obtained for India [14], Italy [24], and Greece [13], but parallels those from Switzerland [21] and Germany [7].

The level of engagement during classes can be seen as an indicator that students wanted to know more about epilepsy. Thus, teachers will encounter a high level of interest amongst students which will facilitate teaching this topic.

It has been proven that epilepsy-related interventions at school such as educational videos or drama had highly significant and long-lasting positive effects upon school children’s knowledge about epilepsy and attitude towards individuals with epilepsy [28]. On the other hand, a study with students from teacher-training colleges using three different methods for education about epilepsy (leaflet reading, colour-slides accompanied by text, and group discussions) found long-term increase in epilepsy-related knowledge, but only short-term positive development concerning attitudes towards people with epilepsy—irrespective of the applied method [29]. Accordingly, it is by no means granted that health education programmes, even if well-intended and carefully planned, have long-lasting educational effects, in particular with respect to attitudes. Here we show that our material successfully and persistently increased knowledge about and attitude towards epilepsy for the majority of students and can thus be recommended to be implemented in school curricula, all the more since only three periods of about 45 min each are needed.

Several studies show that teachers, too, are often not sufficiently informed concerning epilepsy and harbour inadequate attitudes towards people suffering from this disease [24, 28, 30–33], even though they might suddenly face a situation in which they need to act quickly and responsibly when one of their students has a seizure. For example, 14 out of 52 teachers responding to a questionnaire about the relevance of chronic diseases in everyday school life noted that they had been confronted with epilepsy at school [34]. In this respect our material might also help teachers to become more professional in such situations and act as required.

This study may still be regarded as a pilot study and has some limitations which will be addressed in follow-up work. For example, we did not succeed in reducing uncertainty in case of meeting someone with epilepsy with all students. Since many of those argued that they would still feel unsure about what to do in case of witnessing a seizure, even though they theoretically knew how to act, the quiz in our material might be exchanged against a practical session as in first aid courses.

Furthermore, interviews with selected students based upon their answering patterns might help to further understand knowledge and attitude formation (and change due to the intervention) in more detail. This might also help to further improve the teaching material.

## Conclusions

In conclusion, we highly recommend teachers to increase their students’ knowledge about epilepsy and discuss attitudes towards people suffering from this disease. Teaching units such as the one analysed here could contribute to reducing the risk that people with epilepsy are treated disrespectfully and become or feel stigmatized, which may lead to “quality-of-life impairments, including higher than expected rates of unemployment, lower income levels, lower levels of education, reduced rates of marriage, and poorer self-reported health and wellbeing” [33]. Furthermore, such units may help both affected and non-affected people to better understand the various backgrounds for symptoms and how to professionally deal with them.

Implementation of this unit does not require any significant costs, since the videos demonstrating seizure types and presenting case studies are easy to acquire for very little money—in fact, the first one can be loaned out for free. All other material will soon be freely available at the website of the German teacher magazine MNU (see below). However, teachers will have to decide how much time they would want to spend for this topic. It might not be feasible to invest three full lessons as we did in this study. On the other hand, epilepsy is one of the most common neurological diseases and should thus attract sufficient attention at school.

### Key bullet points

- Information about epilepsy is scarce in school textbooks.
- The concept and material tested in this study significantly and persistently increased grade ten high school students’ knowledge about epilepsy.
- After the invention, students’ attitude towards people with epilepsy was significantly and persistently improved.

## Supporting Information

S1 Material Student questionnaire (post-test). Correct answers are printed bold.
(PDF)

S2 Material Data used for statistical analyses. (XLSX)

S1 Table Descriptives for scale scores. CG/TG = control/test group; 1/2/3 = pre-/post-/follow up-test; 1/2/3/4/5 = domains 1–5.
(PDF)
