# Supplementary material for: A Three-Lesson Teaching Unit Significantly Increases High School Students’ Knowledge about Epilepsy and Positively Influences Their Attitude towards This Disease
Source: PLoS One. 2016 Feb 26;11(2):e0150014. doi: 10.1371/journal.pone.0150014 (PMC4771028; doi:10.1371/journal.pone.0150014)
Supplement: S1 Material — Correct answers are printed bold. (PDF) [file pone.0150014.s001.pdf]

Supplemental Material 1.

[Student questionnaire (post-test). Correct answers are printed bold].

Dear Student!
Please fill in this second questionnaire diligently and honestly. This is important to allow us to really improve the teaching unit. Most questions allow for only ONE answer. In case more than one is possible, this is explicitly mentioned. If you are really uncertain with a particular question, you may tick off "I am not sure"

Thank you very much for your assistance with this project!!!

Dr. Uwe Simon, Graz University

Code: First two letters of your first name
+ first two letters of your mother's first name
+ first two letters of your father's first name

(Example: Student's name: ***AL****OIS,*
mother's name: ***SO****NJA,*
father's name ***HU****BERT:*
➔ ***ALSOHU****)*

Code: _____________

Age: _____________

□ female □ male

Mother tongue: ________________

Nationality: __________________

Number of close relatives who work in the medical field (e.g. parents, uncle/aunt as doctor, nurse, etc.): ____________________

Do you know, what ‘epilepsy’ is?
□ yes
□ no
□ I am not sure

If ‘yes’, what is the source of your knowledge? (more than one possibility)
□ by hearsay
□ relatives, friends, acquaintances
□ media
□ others (which?): ____

Write spontaneously down some key words and/or questions concerning ‘epilepsy’!
____
____
____
____

Do you feel uncertain when meeting a person of whom you know that he/she has epilepsy?
□ yes
□ no

If “yes“, why?
____
____
____
____

Is ‘epilepsy’ the same as ‘seizure’?
□ yes
□ **no**
□ I am not sure

Can a person, who has a seizure now and then, work as a teacher?
□ **yes**
□ no
□ I am not sure

Is epilepsy a mental illness?
□ yes
□ **no**
□ I am not sure

Is ‘loss of consciousness’ a possible symptom of a seizure?
□ **yes**
□ no
□ I am not sure

Is it possible to diagnose epilepsy using electroencephalography (EEG)?
□ **yes**
□ no
□ I am not sure

Can a person, who has a seizure now and then, work as a lorry driver?
□ yes
□ **no**
□ I am not sure

Should people with epilepsy be allowed to marry?
□ **yes**
□ no
□ I am not sure

Does epileptic activity during a generalized seizure occur in only one half of the brain?
□ yes
□ **no**
□ I am not sure

Is it always possible to recognize a seizure?
□ yes
□ **no**
□ I am not sure

If 'yes', how?
____
____
____
____

Are people with epilepsy able to live as self-dependent as people without epilepsy?
□ **yes**
□ no
□ I am not sure

Should you always immediately call the ambulance in case you observe a seizure?
□ yes
□ **no**
□ I am not sure

Can someone having had a generalized seizure usually not recall this experience?
□ **yes**
□ no
□ I am not sure

Can a person, who has a seizure now and then, work as a metal worker?
□ yes
□ **no**
□ I am not sure

Is ‘convulsion/twitching of single body parts’ a possible symptom of a seizure?
□ **yes**
□ no
□ I am not sure

Do seizures happening with different people always look the same?
□ yes
□ **no**
□ I am not sure

Are children/adolescents with epilepsy able to attend regular schools?
□ **yes**
□ no
□ I am not sure

Should you place soft cushions below a person having a seizure?
□ **yes**
□ no
□ I am not sure

Are people with epilepsy unpredictable and dangerous?
□ yes
□ **no**
□ I am not sure

Are people with epilepsy allowed to do sport?
□ **yes**
□ no
□ I am not sure

Is ‘absurd behavior’ a possible symptom of a seizure?
□ **yes**
□ no
□ I am not sure

Can a head injury cause a seizure?
□ **yes**
□ no
□ I am not sure

Should women with epilepsy be allowed to become pregnant?
□ **yes**
□ no
□ I am not sure

Should you place a hard object in the mouth of someone having a seizure to protect the tongue, or hold apart the jaw?
□ yes
□ **no**
□ I am not sure

Can a person, who has a seizure now and then, work as a nurse?
□ yes
□ **no**
□ I am not sure

Can an operation help people with epilepsy?
□ **yes**
□ no
□ I am not sure

Is it possible to diagnose epilepsy using magnetic resonance tomography (MRT)?
□ **yes**
□ no
□ I am not sure

Should you wet the face of someone who has a seizure?
□ yes
□ **no**
□ I am not sure

Is epilepsy generally a heritable disease?
□ yes
□ **no**
□ I am not sure

Should you put a person, who has had a seizure with convulsion and loss of consciousness, into recovery position?
□ **yes**
□ no
□ I am not sure

Do people with epilepsy always have seizures regularly?
□ yes
□ **no**
□ I am not sure

Should you try to help a person lay down, if he/she seems to get a seizure soon?
□ **yes**
□ no
□ I am not sure

Are people with epilepsy less intelligent?
□ yes
□ **no**
□ I am not sure

Is 'convulsion/twitching of the whole body' a possible symptom of a seizure?
□ **yes**
□ no
□ I am not sure

Is ‘temperature‘ a possible symptom of a seizure?
□ yes
□ **no**
□ I am not sure

Should you firmly hold a person having a seizure?
□ yes
□ **no**
□ I am not sure

Can teachers expect children with epilepsy to perform equivalently to those without epilepsy?
□ **yes**
□ no
□ I am not sure

Can medication usually help people with epilepsy?
□ **yes**
□ no
□ I am not sure

Is ‘falling down‘ a possible symptom of a seizure?
□ **yes**
□ no
□ I am not sure

Do people with epilepsy always need a lifelong treatment?
□ yes
□ **no**
□ I am not sure

Can shortage of sleep cause a seizure?
□ **yes**
□ no
□ I am not sure

Should you remove sharp objects, edged furniture etc., when you observe a seizure?
□ **yes**
□ no
□ I am not sure

Can a person, who has a seizure now and then, work as a police officer?
□ yes
□ **no**
□ I am not sure

Would you begin a love relationship with someone you know has epilepsy?
□ **yes**
□ no
□ I am not sure

Can extensive drinking of alcohol cause a seizure?
□ **yes**
□ no
□ I am not sure

And now some questions concerning the last lessons:

Note down some key words you think are important to describe epilepsy!
____
____
____
____

During the lessons you have watched seizures in the videos. How did you feel?
____
____
____
____

Was the topic presented in an interesting way?
□ yes
□ no

Was the logic of the teaching unit clear to you?
□ yes
□ no

Was the material comprehensible, helpful, and clear to you?
□ yes
□ no

What could have been better?
____
____
____
____

Is there anything you would include in the teaching unit which has not been dealt with?

____
____
____
____

Reflecting on the lessons about ‘epilepsy: What was new for you?

____
____
____
____

Has the teaching unit changed your attitude towards people with epilepsy?

□ yes
□ no

If ‘yes’, how?

____
____
____
____

Once again: Many thanks for your efforts!
