# Supplementary material for: A Three-Lesson Teaching Unit Significantly Increases High School Students’ Knowledge about Epilepsy and Positively Influences Their Attitude towards This Disease
Source: PLoS One. 2016 Feb 26;11(2):e0150014. doi: 10.1371/journal.pone.0150014 (PMC4771028; doi:10.1371/journal.pone.0150014)
Supplement: S1 Table — CG/TG = control/test group; 1/2/3 = pre-/post-/follow up-test; 1/2/3/4/5 = domains 1–5. (PDF) [file pone.0150014.s003.pdf]

Supplementary Table 1. Descriptives for scale scores. CG/TG = control/test group; 1/2/3 = pre-/post-/follow up-test; 1/2/3/4/5 = domains 1 – 5.

Group * time * domain			Mean	Std. Error	95% Confidence Interval Lower Bound	Upper Bound
CG	1	1	,468	,025	,419	,517
		2	,665	,027	,611	,719
		3	,272	,027	,219	,326
		4	,755	,028	,699	,811
		5	,486	,026	,435	,537
	2	1	,472	,020	,432	,512
		2	,663	,024	,616	,710
		3	,251	,022	,208	,295
		4	,753	,022	,710	,796
		5	,524	,020	,484	,564
	3	1	,471	,021	,430	,512
		2	,623	,025	,573	,673
		3	,235	,026	,184	,287
		4	,769	,021	,728	,809
		5	,532	,023	,487	,577
TG	1	1	,430	,019	,392	,468
		2	,588	,021	,546	,630
		3	,240	,021	,198	,281
		4	,747	,022	,703	,790
		5	,402	,020	,363	,442
	2	1	,813	,016	,782	,844
		2	,807	,018	,771	,843
		3	,898	,017	,865	,932
		4	,931	,017	,897	,964
		5	,893	,016	,862	,924
	3	1	,779	,016	,747	,811
		2	,756	,019	,718	,794
		3	,805	,020	,765	,845
		4	,927	,016	,895	,958
		5	,870	,018	,835	,905
